# Cell-penetrating peptides as facilitators of cargo-specific nanocarrier-based drug delivery

**DOI:** 10.1039/d5nr00617a

**Published:** 2025-08-14

**Authors:** Ashleigh M. Jankowski, Matthew A. Ensign, Katharina Maisel

**Affiliations:** a Fischell Department of Bioengineering, University of Maryland College Park MD USA maiselka@umd.edu

## Abstract

Nanocarriers (NCs) have emerged as a prime tool for drug delivery, with their highly tunable properties allowing for targeting down to the organelle level. For successful drug delivery, NCs of all administration routes face challenges to reaching target sites in the form of various biological barriers. A prominent one of these barriers is cellular uptake. Conjugation of cell-penetrating peptides (CPPs) to NC surfaces has been found to overcome this barrier, as such enhancing percent accumulation in the target site. Recently, novel CPPs have been found to enhance efficacy of NC platforms *via* their ability to aid in overcoming additional barriers such as the mucus layer, immune clearance, and non-specific accumulation. The design of NC–CPP drug delivery platforms involves consideration to the synergy between the NC type, cargo, target cell, and CPP. This review dives into recent developments of these platforms and into novel CPPs for NC drug delivery, also highlighting areas of future research. We cover a broad swath of the field, touching on novel instances and trends investigating the use of newly designed CPPs modulated for recognizing specific ligands for active targeting, NC–CPP combinations which tackle biological barriers such as the blood–brain barrier, and those with unique cargos from nucleic acids to proteins.

## Abstract 2

Cell penetrating peptides have emerged as a prime tool for enhancing nanocarrier barrier penetration and targeted drug delivery outcomes. Illustration made using BioRender. The graphical abstract was created in BioRender. Maisel, K. (2025) https://BioRender.com/iouxsm2.

## Graphical abstract

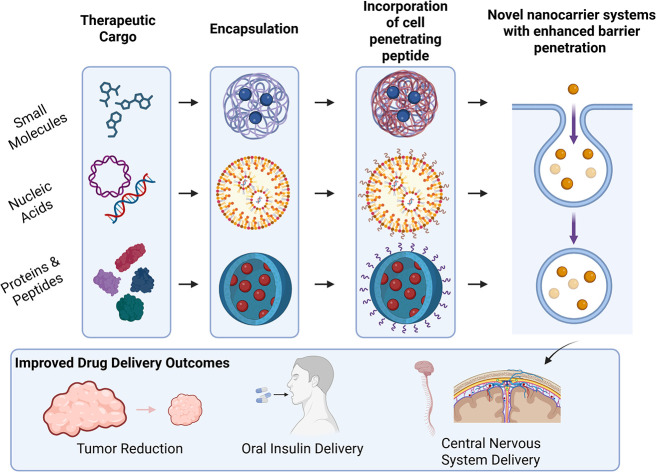

## 1. Introduction

Nanocarriers (NCs) have emerged as a top drug delivery vehicle due to their highly tunable properties allowing for targeting down to the organelle level, crossing of complex biological barriers, high surface area to volume ratio, increased therapeutic efficacy, and decreased off-target effects.^1^ While originally developed with a focus on chemotherapeutic delivery, applications of NCs have stretched beyond cancer to include a vast array of diseases, including rare and chronic diseases, opening doors for new and improved treatment options to improve their clinical outcomes.^2^ Despite significant promise in this work and early successes, there is still a need for more research and development: as of 2021, just over 25 drug-loaded s nanomedicine treatment platforms have been approved by the FDA and/or EMA, but all of them have limitations.^2,3^ A primary reason for this is that despite significant efforts, targeting itself is still an issue. A comprehensive study found that in nanomedicines for cancer treatment, the average percent of administered NCs that make it to the target site is 0.7%, due to interactions with a wide variety of biological barriers, including immune clearance, nonspecific uptake, endosomal degradation, and cellular efflux, to name a few.^4^ This indicates a need for systems with higher targeting yield, to in turn enhance clinical efficacy and reduce required dosages. Cell-penetrating peptides (CPPs) are now emerging as a popular peptide class which address this need. CPPs improve NC transport across biological barriers by improving nonspecific penetration at the cellular level, and in certain cases, passive and/or active targeting at the systemic level, enhancing the percentage of administered particles which make it to the site of action.

CPPs are a diverse class of short (5–40 amino acid s(AA), long) and often linear peptides that vary greatly in size, charge, sequence, balance of hydrophobicity and hydrophilicity, and origin (natural or hybrid/synthetic).^5–7^ The first CPP discovered, the transcription-transactivating protein of HIV-1, or TAT, was identified in 1988 after the domain was observed penetrating the cell membrane and nucleus. TAT was then conjugated to small molecule drugs and biologics to enhance uptake into cells and across biological barriers, and more recently to improve uptake of larger cargos, like NCs. A significant portion of newer CPPs is derived from TAT, using the key uptake sequence 47-YGRKKRRQRRR-57.^8^ A wide array of natural and synthetic CPPs has also been created and used to enhance intracellular drug delivery. This diverse family of peptides can directly translocate through cell membranes or associate with membrane lipids/receptors to improve cargo uptake. Notably, CPP-conjugated delivery systems exhibit first-order kinetics with short half-lives and can have low cytotoxicity.^9,10^ Conjugating CPPs to drug-loaded NCs leverages both the improved barrier penetration capabilities of the peptide and the stability of the NC, which can reduce drug degradation outside the cell. Novel sequences, rationally designed and synthesized specifically for NC formulation, include sequences with domains that intercalate within the lipid bilayer of liposomes or peptides that themselves act as NCs. While other notable reviews have delved into the efficacy of CPPs for NC delivery before, the focus of these are largely solely chemotherapeutics, and/or do not emphasize the key synergistic features of these novel cargo–CPP–NC systems which allow for barrier penetration and accumulation in target sites.^11–13^ This review aims to cover the scope of these novel CPPs, as well as the uses of well-established CPPs for NC-based drug delivery. We highlight CPP–NC research, which investigates the relationship between CPPs, NCs, and cargo, and its synergistic effects on enhancing delivery of small molecules, nucleic acids, and proteins to their target sites in order to provide insights into design on a system level. Key examples are highlighted below, in Table 1. We also discuss areas where more research is needed.

**Table 1:** Key literature examples

Cargo	Novelty area	Significant breakthrough	Key literature examples
Small molecule chemotherapy + cancer treatment	CPPs for enhanced tumor targeting and retention	Conjugation of specific CPPs overcomes hallmarks of chemotherapeutic resistance, including efflux and Epithelial–mesenchymal transition mediated-resistance	Bhatt, *ACS Appl. Bio Mater.* (2020);^39^ Ryu, *Int. J. Mol. Sci.* (2021);^40^ Lo, *Theranostics* (2020)^41^
		CPPs constraining RGD motifs can be used to create tumor specificity in NPs	Chen, *Biomed. Pharmacother.* (2022);^43^ Yamada, *ACS Omega* (2023);^44^ Li, *Int. J. Nanomed* (2021);^45^ Fan, *Eur. J. Pharm. Biopharm.* (2020)^46^
		Conjugation of CPPs containing a R/KXXR/K-specific motif or novel TAT-AT7 CPP enhances tumor specificity through NRP-1 binding	Ma, *ACS Appl. Bio. Mater.* (2021);^49^ Wang, *Int. J. Mol. Sci.* (2023);^50^ Lu, *Int. J. Nanomed.* (2020)^57^
	CPP–NC–cargo systems for controlled on-site payload release	Conjugation of modified CPPs enhances cell penetration exclusively in acidic tumor microenvironment	Zhang, *Front. Bioeng. Bioetchnol.* (2023);^54^ Zhang, *Langmuir* (2019);^55^ Zhang, *Pharmaceutics* (2022)^56^
Nucleic acids	Arginine-based CPPs	RGD in cyclic (cRGD) conformation enhances RNA tumor cell uptake	Nai, *Mol. Ther. Nucleic Acids* (2022);^58^ Gao, *Int. J. Nanomed*. (2021)^59^
	Transfection-oriented	PepFect/NickFect CPPs enhance transfection	Kiisholts, *Pharmaceutics* (2021);^60^ Abdelhamid, *Microporous Macroporous Mater.* (2020)^61^
		Amphiphilic and Hydrophilic CPP sequence modifications enhance uptake and expression	Deshayes, *BBA* - *Biomembranes* (2020);^62^ Sugimoto, *Drug Deliv.* (2023);^63^ Bazaz, *Biomedicine*, (2021);^64^ Gomes dos Reis, *Drug Dev. Ind. Pharm*. (2020)^65^
Proteins and peptides	Cancer treatment	CPPSs enhance cytosolic availability of protein drugs	Qian, *Theranostics* (2019);^66^ Baehr, *Biomaterials* (2021);^67^ Lee, *ACS Nano* (2025)^73^
		Novel CPPs aid in overcoming barriers to enhance antigen presentation	Liu, *Biomater. Sci.* (2019);^71^ Mohammadi, *J. Cell Physiol.* (2021)^72^
	Diabetes treatment	Electrostatic interactions between drug cargo and CPP can affect barrier permeation and percent loading	de Souza Von Zuben, *Colloids Surf.*, *A* (2021);^70^ Tan, *Biomateri Sci*. (2019);^68^ Li, *J. Pharm. Sci.*, (2021);^69^ Tan, *Mol. Pharm.* (2020)^26^
Co-delivery	Dual small molecule	Dual incorporation of drugs in a CPP-conjugated NC improved chemotherapeutic efficacy *via* increased tumor sensitivity	Zhao, *JCR* (2023);^75^ Liu, *ACS Biomater. Sci. Eng.* (2022)^76^
	Small molecule - nucleic acid	Novel CPP domain enhances endosomal escape of NCs	Zhao, *Nanobiotechnology* (2022)^77^

## 2. Overview and benefits of cell-penetrating peptide technologies

### 2.1 Challenges to systemic drug delivery

Small molecule drugs, natural or synthetic organic compounds that are <500 Da,^14^ in particular face an array of challenges for systemic drug delivery. Free drugs tend to be quickly cleared and accumulate in off-target organs, yielding significant side effects, and many are only taken up by either simple diffusion or require transporters, which reduces intracellular bioavailability.^15,16^ NC encapsulation of small molecules has been found to address some of these issues, including increasing circulation half-life, drug solubility (for hydrophobic molecules), and bioavailability, all while reducing off-target accumulation by providing a platform that can be modified for localized targeting.^15^ However, NC drug delivery comes with its own set of unique challenges, including the blood endothelial barrier, which limits NC extravasation into the interstitial tissue, the charged extracellular matrix (ECM) that can trap oppositely charged NCs, and plasma membranes that can only be crossed *via* efficient interactions with membrane proteins or the membrane itself.^15^ Conjugating CPPs to NCs can address these issues, improving cell penetration and uptake. Though conventional linear CPPs lack specificity and can be degraded by proteases or trapped within lysosomes upon endosomal uptake into cells,^17^ modification of their sequences and structures to circumvent these issues and aid in cargo loading is ongoing.^18^

### 2.2 Types of CPPs

The term CPP is broad and encompasses a variety of sequences with distinct structures that have been shown to enhance cell penetration. Each sequence can be categorized in terms of its amphiphilicity and derivation (natural *vs.* synthetic). CPPs are commonly cationic peptides that contain mostly positively charged amino acids, such as arginine and lysine. Amphiphilic CPPs contain both hydrophilic and hydrophobic residues and often adopt secondary α-helical structures. The most common CPPs described in literature that are used to enhance the cellular uptake of conjugated NCs are TAT (YGRKKRRQRRR), a cationic peptide derived from the HIV-1 virus; penetratin (RQIKIWFQNRRMKWKKGG), a secondary amphipathic peptide derived from the *Antennapedia* homeodomain of *Drosophila;* octa-arginine, a synthetic, highly cationic oligopeptide; and transportan (GWTLNS/AGYLLGKINLKALAALAKKIL), a chimeric amphipathic peptide derived from the neuropeptide galanin and the toxin mastoparan.^10,19,20^

CPP structure and amino acid sequence dictate their ability to deliver conjugated drugs/NCs. A key example of this was recently observed by Gessner *et al.*, who saw that swapping positively charged arginines and lysines with alanine in an arginine-rich amphiphilic sC18 peptide reduced internalization, except for when swapped at position 15, which increased both charge and uptake.^7^ Similarly, Habault *et al.* observed that replacement of aspartic acid with arginine in the amphiphilic hAP10 CPP significantly increased penetration efficiency, demonstrating 50% higher penetration than TAT.^6^ These studies indicate that secondary peptide structure is essential for interacting with biological membranes. On the other hand, certain peptide modifications have negligible impacts on cell penetration: deletion of the 47^th^ amino acid in TAT had no significant effect on its translocation, and switching C–N or C–C linking (effectively flipping the peptide sequence) of antigens to CPP had no significant effect on uptake of peptide vaccines.^5,9^ While these works represent key steps in understanding the structure–function relationships of CPPs, there is still a need to elucidate this relationship further, particularly in the context of NC–CPP conjugation.

### 2.3 Cellular uptake mechanisms

Two categories of internalization mechanisms utilized by CPP–NCs have been observed: direct translocation, an energy-independent process that relies on destabilizing the cell membrane temporarily to allow translocation, and endocytosis, an energy-dependent process that relies on interactions with membrane lipids or proteins. These mechanisms are further subdivided into several pathways. One of these pathways is known as micropinocytosis, of which clathrin- and caveolae-mediated endocytosis are prime examples. Clathrin-mediated endocytosis is mediated by multiple low-affinity protein–protein and protein–lipid interactions that lead to the formation of clathrin-coated pits that invaginate and pinch off into intracellular endocytic vesicles. Caveolae-mediated endocytosis is similar in that caveolin and accessory proteins are recruited during an invagination of the cell membrane that pinches off and traffics to an early endosome in the cell.^21^ Macropinocytosis, different from micropinocytosis like clathrin- or caveolin-mediated endocytosis, occurs when an actin-driven expansion of the cell membrane encloses extracellular fluid and folds back onto the cell membrane into a macropinosome, which matures into an early endosome.^21^ Although CPPs have been observed to enhance NC cargo uptake *via* these uptake mechanisms, there is a lack of understanding as to what is causing these interactions/mechanisms at the cellular level. There is a need for further elucidation on the relationship between cell type, CPP sequence, sequence modification (cyclization), molecule conjugation receptor expression, peptide concentration, and the binding strategy of the cargo, as all of these factors affect CPP–NC internalization.^19,20,22–24^ Some notable trends in cellular uptake mechanisms have been observed; however, most CPPs have been found to leverage multiple mechanisms.

**Fig. 1:**
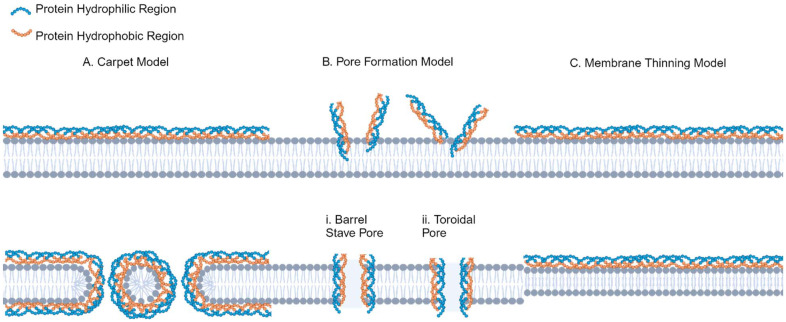
CPP penetration models. (A) carpet model, (B) pore formation model; (i) barrel stave pore, (ii) toroidal pore, (C) membrane-thinning model. Illustration made using BioRender. Created in BioRender. Maisel, K. (2025) https://BioRender.com/l63e761.

Cationic peptides are translocated *via* energy-independent interactions with anionic membrane components, causing membrane instability. Three primary models have been proposed: the carpet model (Fig. 1A), pore formation model (Fig. 1B), and the membrane-thinning model (Fig. 1C). In the carpet model, hydrophobic residues embed into the membrane and hydrophilic sites orient to the polar face, causing a reorganization of the membrane that disrupts the bilayer and allows the CPP to enter.^25^ In the pore formation model, amphiphilic peptides intercalate with the cell membrane and form direct pores that allow cargo to penetrate. The membrane-thinning effect is observed when negatively charged lipids in the outer membrane interact with cationic residues, allowing the CPP to intercalate within the laterally rearranged membrane.^19^

Arginine has been found to be a crucial residue for achieving membrane thinning, as it electrostatically interacts with the plasma membrane to penetrate it without active transport. Heparin sulfate proteoglycan (HSPG), an anionic membrane component, appears to be a key component involved in the uptake of arginine-rich peptides and arginine-based CPP–NCs. This was recently observed in human glioblastoma U87 spheroids incubated with micellar NCs conjugated to octa-arginine, in enteroid models incubated with mesoporous silica NCs bound with PLA-PEG-protamine (VSRRRRRRGGRRRRC), and binding studies investigating membrane protein binding with arginine in CPPs.^6,24,26^

Amphiphilic CPPs can directly translocate across or enter the cell through endocytosis. The former mechanism requires the formation of an inverted micelle in the plasma membrane (due to peptide–lipid interactions). Then, pore formation occurs in a “barrel-stave” (hydrophilic residues form the core of a large pore) or “toroidal” (lipids bend to interact with CPPs at their head groups) model upon reaching a certain peptide concentration (Fig. 1B).^19^ This method depends on hydrophilic regions in the sequence that stabilize the peptide when interacting with membrane proteins, and there is an optimum proportion of hydrophilicity and hydrophobicity as the strength of the protein–peptide interactions can either prevent translocation or sufficiently destabilize the membrane to allow the peptide to pass through.^27^ Endocytic mechanisms that translocate amphipathic peptides include clathrin- and caveolae-mediated endocytosis and macropinocytosis. For endocytic uptake in general, induction of membrane curvature by amphiphilic sequences promotes endocytic uptake *via* formation of an endocytic cup and then vesicle. This appears to be dependent at least in part on the secondary structure of the peptide; for an artificial nine-residue peptide (R6W3, RRWWRRWRR-amide), when α-helical structures and facial amphiphilicity were lost, the improved uptake was lost.^28^ Beyond this, specific residues in amphiphilic peptides appear to promote certain endocytic mechanisms. Proline-rich amphipathic peptides are thought to interact with ECM glycosaminoglycans, and studies co-delivering cholera toxin found that uptake with these CPPs depended on caveolae-mediated endocytosis.^29^

Combination of CPPs with NCs has also been found to influence uptake. Octa-arginine conjugation to polyethylene glycol-conjugated (PEGylated) liposomes, with a 1% peptide density and containing short serine-glycine repeat spacers, had a primary dependence on clathrin-mediated endocytosis in glioma cells. in another study, macropinocytosis was the predominant uptake mechanism used by U87 spheroids when incubated with elastin-like polypeptide NCs conjugated with octa-arginine.^24^ These studies demonstrate that the introduction of NCs further complicates and affects the type of uptake, as uptake depends on the physiochemical properties of both the peptide and the cargo, and also that uptake mechanisms may be dependent on the type of cellular barrier that the CPP–NC is facing.^29^

**Fig. 2:**
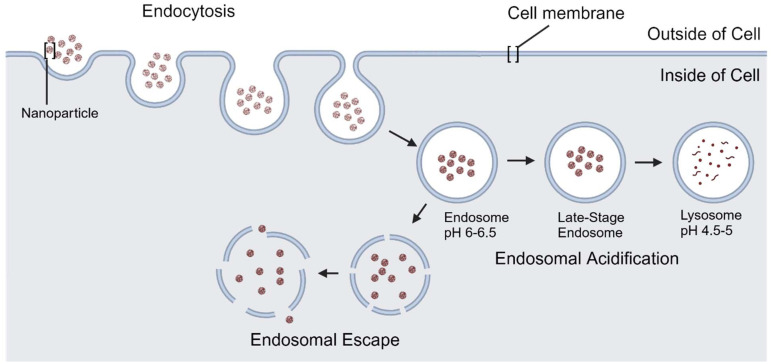
Diagram of endocytosis, endosomal acidification, and endosomal escape. Materials taken up by an endosome will be degraded once an endosome has acidified and transitioned into a lysosome, necessitating endosomal escape for intracellular delivery. Illustration made using BioRender. Created in BioRender. Maisel, K. (2025) https://BioRender.com/s81a403.

Once endocytosed peptides enter the cell, the next barrier to delivery is endosomal escape. Upon internalization, endocytosed NCs are contained within endosomes, which may mature into late endosomes and then lysosomes that degrade the CPP and its cargo (Fig. 2). If the delivery system cannot escape the endosome, it will be rendered nonfunctional (unless it is not biodegradable). Cationic residues have been observed facilitating endosomal escape by inducing endocytic vesicle membrane leakage or disruption.

Certain CPPs, then, can serve dual-purposes: cellular uptake and endosomal escape. For example, TAT induces endosomal leakage by interacting with anionic phospholipids, and oligo-arginines form ion pairs with lipids to partition the membrane.^19^ The presence of bis(monoacylglycero)phosphate (BMP), which is uniquely abundant in late endosomes, and the distance between lipid bilayers in the endosomal wall are the most important factors in CPP-mediated endosomal escape.^30^ To translocate out of the endosome, cationic CPPs must be encapsulated at sites where the lipid bilayers are in close contact so that BMP can interact with peptide chains, promoting encapsulation. This suggests that interactions between cargo and peptide do not affect endosomal escape.^30^ However, recent work is in direct contradiction to this: Patel *et al.* incubated 10T1/2, HepG2, HeLa, and HEK cells with eight GFP–peptide conjugates containing linear, cyclic, or hemagglutinin-conjugated TAT, linear or cyclic octa-arginine, transportan, penetratin, or Xentry, and observed no significant endosomal escape.^23^ Conjugation in general may reduce escape as other researchers have shown that nonlinear, low molecular weight PEG spacers can attach to the wall of the endosome and prevent associations between peptides and lipids. It is possible that the efficacy and method of endosomal escape may vary with CPP type and sequence, cargo, and other factors, which would explain the differences in these findings. However, the interactions between cargo, NC, and externally conjugated components are not fully understood for most designed, complex CPP–NCs.^22^ This knowledge gap is due to a lack of investigation as most studies focus on improving therapeutic outcomes and do not include experiments measuring colocalization of CPP–NCs with endosomes, let alone the mechanisms by which the NCs exit these vesicles.

### 2.4 Potential toxicity of CPP-NPs

With any drug, toxicological impacts are an obvious concern. Reports following administration of CPP conjugates in preclinical studies have demonstrated off-target and systemic toxicity in some cases. Cationic CPPs may be more prone to causing toxicity due to their strong penetrative capacity and positive charge, while electroneutral CPPs performed better *in vivo*.^31^ For example, a recent study found that nuclear entry of arginine-rich CPPs can displace RNA- and DNA-binding proteins from chromatin and mRNA, impairing RNA processing and DNA repair.^32^ However, this is not a hard rule as TAT, one of the most commonly used CPPs, is relatively nontoxic compared to other cationic CPPs.^31^

Most studies have reported that, overall, CPP-mediated toxicity is low but varies with the physicochemical properties of the CPP and its cargo.^33^ CPP–NCs engineered with these potential challenges in mind have been promising. In one study, the combination of a novel lactoferrin-derived CPP with a biodegradable PLGA nanoparticle caused no impacts on mitochondrial activity or membrane permeability when administered to two lung epithelial cell lines.^34^ Compared to viral vectors, which are often used to intracellularly deliver macromolecules, CPPs demonstrate high delivery efficiency without notable cytotoxicity.^35^ Thus far, no CPP-conjugated drugs have been approved by the FDA and the most significant limitations of these drugs is their short duration of action, low *in vivo* stability, and lack of specificity if not designed with a specific target in mind.^33,36^ However, as described in the rest of this review, recent data is promising, and researchers have developed ways to overcome a significant number of these issues.

## 3. CPPs for small-molecule delivery

### 3.1 CPPs in NC-based chemotherapeutic delivery

The most common small molecule drugs delivered by CPP–NCs are chemotherapeutics. Conventional cancer treatment relies on chemotherapeutics with cytostatic or cytotoxic properties that kill cancer cells, but these drugs cause widespread systemic toxicity when administered freely. Tumors have also been observed to develop resistance against chemotherapeutics, such as *via* efflux pumps that remove endocytosed drugs.^37^ In multidrug-resistant cancer cell lines like NCI/ADR-RES, the *ABCB1* gene encoding the P-glycoprotein (Pgp) efflux pump is overexpressed, reducing accumulation of chemotherapeutics like doxorubicin, paclitaxel, etoposide, and irinotecan.^38^ Novel CPP–NC formulations have been found to circumvent these issues, yielding better prognostic outcomes. In one study, conjugation of polyarginine CPP (R8) and vitamin E succinate, P-glycoprotein inhibitor, to paclitaxel-loaded poly(amidoamine) dendrimers significantly increased melanoma cell uptake compared to free drug and prevented efflux of paclitaxel, leading to an increased overall tumor volume reduction in B16-F10 tumor-bearing mice.^39^ Recent work also suggests that coupling CPPs to chemotherapeutics can prevent ABC-transport-mediated efflux, improving drug retention within the tumor.^40^ For example, a thermosensitive elastin-like polypeptide was conjugated to Dox-TAT with a matrix metalloproteinase substrate, generating a delivery system that aggregates in hyperthermic tumors and releases CPP-Dox upon degradation of the linker.^40^ The CPP-Dox conjugate demonstrated improved killing of Dox-resistant cancer cells *in vitro*. Overcoming chemotherapeutic efflux is a pertinent challenge in taking the next step toward improved cancer treatments, and while early research indicates CPP conjugation to chemotherapy-loaded NCs is a viable means of addressing this challenge, further investigation of the specific uptake mechanisms at play and how utilization of these mechanisms circumvents efflux is still needed in order to design and fine-tune novel CPPs to maximize these effects.

Epithelial–mesenchymal transition (EMT) is known to contribute to elevated migration and invasion and confer resistance to chemotherapeutics like irinotecan. Thus, delivery systems combining CPPs with molecules designed to inhibit this transition are also of interest. Suppression of the EMT was achieved by loading miR-200, which inhibits zinc finger E-box binding homeobox 1 (a key driver of EMT that suppresses miR-200 when overexpressed) together with irinotecan, in CPP-decorated lipid nanoparticles (LNP). In this study, the LNPs were decorated with a C peptide for cell penetration, N peptide for targeting to nerve/glial antigen 2 overexpressed on neovascular tumors, and a mitochondrion-localizing M peptide with pro-apoptotic KLA residues. This formulation was observed to enhance cancer cell killing in an oral squamous cell carcinoma mouse model.^41,42^ The combination of CPPs with chemotherapeutics and strategies to overcome known barriers to cancer cell delivery presents a valuable opportunity to improve the efficacy of existing cancer treatments. These promising results should encourage future *in vivo* studies using similar formulations.

Synthetic CPPs and sequences inspired by natural peptides that bind to integrins have been explored in the context of chemotherapeutic-loaded NCs. Integrins are receptors for extracellular matrix proteins that are generally involved in cell adhesion, differentiation, and survival. Some integrins are upregulated during tumor progression (*i.e.*, αvβ3 and αvβ5)^43^ and can be targeted using the RGD motif [comprised of arginine (R), glycine (G), and aspartic acid (D)] which binds to multiple integrins.^44^ iRGD, a cyclic, tumor-homing modified version of RGD has an exposed arginine at the C-terminal end, promoting membrane penetration and tumor-specificity. Li H. *et al.* decorated 10-hydroxycamptothecin and indocyanine green-loaded phase transformation liposomes with iRGD and combined them with low intensity ultrasound to promote tumor cell binding and uptake and release of 10-hydroxycamptothecin, promoting apoptosis.^45^ To enhance CPP function, a novel linear RGD-CPP variation, Pm45, was synthesized. This variant was shown to integrate into erythrocyte-derived vesicles due to its lipid-mimicking hydrophobic helical tail and anionic headgroup linked by the RGD domain. The decorated vesicles specifically bound to MDA-MB-231 cells expressing αvβ3, which caused enhanced accumulation of loaded doxorubicin (DOX) in mouse tumor xenografts compared to non-targeted liposomes.^46^

Another accessible target that differentiates tumors from healthy tissues is neuropilin-1 (NRP-1), a receptor found on immune and endothelial cells that is overexpressed by many cancer cells and involved in angiogenesis.^47,48^ Peptides with a R/KXXR/K-specific motif [comprised of arginine or lysine (K) and two other amino acids] bind specifically to this receptor, promoting cancer cell specificity and uptake.^49^ Wang L. *et al.* designed another novel CPP for NRP-1 targeting, the cell-penetrating peptide TAR, a fusion peptide of the commonly used cell-penetrating TAT and the NRP-1-targeting A7R (ATWLPPR) peptide.^50^ When fused to paclitaxel through an acid and esterase sensitive bond, drug release was enhanced, and increased cellular uptake through receptor-mediated endocytosis was observed in HUVEC cells. Deep penetration into a tumor spheroid was also observed and inhibited tumor growth.^50^

CPP modification to introduce a targeting component has also been investigated for treating central nervous system (CNS) cancers, such as gliomas. CNS delivery presents an additional challenge to small-molecule drug delivery due to the blood brain barrier (BBB). An intact BBB is extremely effective at preventing transport of drugs from circulation and restricts the passage of more than 98% of small-molecule drugs.^51^ To overcome this, CPP–NCs with targeting components are being investigated. A recent study used intranasally-administered TAT-conjugated polymer micelles and stearoyl-modified bioactive-peptide bombesin micelles to deliver camptothecin to mice with C6 glioma grafts. These formulations successfully circumvented the BBB, allowing the loaded drugs to accumulate in the parenchyma *via* the perineuronal space.^52^ Incorporation of the bioactive peptide bombesin improved the selectivity of the vehicle by binding to the gastrin-releasing peptide receptor abundantly expressed on glioblastomas, yielding improved accumulation in tumor tissue and overall improved survival.^52^ The incorporation of sequence motifs with known targeting capabilities into well-established CPPs represents a key opportunity to develop CPPs that enhance both tumor-targeting and tumor-cell uptake, improving therapeutic outcomes.

### 3.2 Stimulus-responsive CPPs in small-molecule drug delivery

Many naturally derived and synthetic CPPs, such as penetratin and polyarginine sequences, penetrate cells nonspecifically and can therefore cause off-target effects. CPP–NC systems may be designed to respond to specific stimuli by undergoing conformational changes or chemical reactions that facilitate targeted delivery. These stimuli could be endogenous environmental conditions like the acidic pH of the tumor microenvironment or late endosome, the presence of an enzyme targeting a degradable linkage or polymer, or hypoxia. Alternatively, exogenous stimuli, such as light and ultrasound, could trigger a conformational change in the NC that leads to the release of loaded drugs, which could also be responsive to various stimuli. The rational design of these systems can generate formulations that perform significantly better than free drug.

Shi H. *et al.* synthesized molecularly imprinted polymer (MIP) NCs for treating subcutaneously implanted 4T1 (murine mammary carcinoma) tumors in mice. These NCs are formed by polymerizing organosilane on a Fe_3_O_4_ template, and then decorating them with DOX, the cell-penetrating peptide GAFPHR, and an aggregation-induced isothiocyanate photosensitizer (TBTCP-CA).^53^ The result is a NC that exhibits pH responsive release of DOX at endosomal pH and stimulates reactive oxygen species (ROS) production when irradiated. Conjugation with a CPP improved tumor uptake in a xenograft mouse model, leading to significant reductions in tumor volume. This same principle was leveraged for photodynamic therapy by encapsulating the photosensitizer chlorin e6 in a mixed polymeric micelle, which also contained the hypoxia-activated prodrug tirapazamine (TPZ) and presented dimethyl maleate-masked (DA-masked) TAT peptide.^54^ The acidic tumor microenvironment unmasked the peptides to enhance tumor penetration. Irradiation with red light then induced hypoxia to disassemble the NC and convert TPZ to cytotoxic radicals, leading to inhibition of tumor growth in an *in vivo* breast-cancer model. Stimuli-responsive CPP–NC systems represent a significant opportunity to continue to reduce off-target accumulation and effects, enhancing overall efficacy.

Self-assembling peptide-based NCs, comprised of a peptide sequence containing both a cell-penetrating motif and a stimulus-responsive motif, can create targeted NCs that release theircargo under desired conditions. Zhang L. *et al.* designed the NP1 peptide (stearyl-HHHHHHHHHHHHHHHH-RRRRRRRR-NH2), which self-assembles into a globular structure with the arginine sequence exposed at neutral and basic pH. NP1 then undergoes passive cellular uptake, disassociates in the acidic pH of the tumor cell, and releases ellipticine (an anticancer agent) into the cytosol.^55^ Similarly, Zhang M. *et al.* developed an enzyme-responsive, self-assembling peptide-based NC using a core of repeating GFLG oligopeptides highly specific to cathepsin B for cytoplasmic DOX release. This strategic peptide design leverages arginine for enhanced cell penetration and histidine for facilitating lysosomal escape through proton buffering. Compared to free DOX, the viability of HeLa cells and spheroids was significantly reduced.^56^

The multi-functionality of CPPs for small molecule chemotherapeutic delivery is just beginning to be explored, with emphasis on modification of sequences for targeting and controlled release purposes. The modification of CPPs for this purpose, combined with their primary function of enhanced cellular uptake, represents a prime opportunity for highly targeted, highly effective drug delivery platforms.

## 4. CPPs for nucleic acid cargo

Delivery of nucleic acids (NAs), specifically genetic material, is used for transfection and genetic modification in a variety of diseases with known genetic mutations, including cancer. For successful functionality, these NAs must be delivered to the cytosol or within the nucleus, depending on the type of genetic material being delivered and the intended mechanism of action. NCs are the preferred vehicle to deliver NAs by shielding from the external environment to reduce degradation, toxicity, and immunogenicity. Recent work has shown that external conjugation of CPPs can enhance NP uptake and potentially the targeting capabilities of NCs, enhancing transfection and therapeutic outcomes overall. Arginine-rich CPPs have been primarily used for NA delivery, since arginine facilitates endosomal escape *via* the proton sponge effect due to its strong positive charge. Recent research has explored modifications to arginine-rich CPPs to add functionality, including enhanced cell-specific targeting, transfection, and bioavailability.

### 4.1 Arginine-containing CPPs for tumor targeting

Enhancing CPPs through modifications to their structure, either by addition of novel groups or specific sequences to change surface chemistry (*i.e.*, introduction of hydrophobic or amphiphilic groups) to target specific tissues or other sites (*i.e.*, tumors), greatly enhances the efficacy of NC-delivered nucleic acid cargo. Targeting and uptake capabilities of polyethylenimine (PEI) NCs, loaded with an angiogenesis-inhibiting secretory endostatin gene, were significantly enhanced by the addition of TAT-AT7, a novel protein with high affinity for VEGFR-2 and NRP-1 receptors. NCs with TAT-AT7 were found to have better receptor binding than AT7 alone, and better endothelial cell uptake than TAT alone, indicating synergistic effects and allowing for high selectivity and uptake in glioma.^57^ Lu *et al.* found that a (PEI)-TAT-AT7 NC delivering an angiogenesis-inhibiting secretory endostatin gene had enhanced suppression of tube formation and endothelial cell migration, inhibited glioma growth, and reduced microvasculature formation in U87 glioma mice.^57^ Cyclic RGD (cRGD)–NC conjugation has also been found to enhance tumor targeting and tumor cell uptake of RNA-delivering NCs. In investigating the use of liposomes and modified arginine-rich c-terminal cystine cRGD CPPs for improving intracellular delivery of siRNA, Nai *et al.* found that while CPPs alone could be conjugated to siRNA to improve uptake, siRNA encapsulated in liposomes with externally conjugated CPP best improved uptake and tumor cell targeting, suggesting synergistic effects.^58^ Similar results were achieved with a PEGylated lipid NC conjugated to cRGD and polyarginine CPPs, which significantly improved tumor targeting compared to naked LNPs.^59^ However, more work needs to be done to investigate if there were synergistic effects of dual cRGD and PEG conjugation. Another example of an arginine-based CPP approach is a glycopolymer–arginine-rich peptide conjugate designed by Peng *et al.* that condenses siRNA, forming a polyplex. The NC was shown to localize in the cytoplasm of HeLa (cervical cancer) cells and demonstrate superior EGFR silencing compared to lipofectamine 3000, a commercially available transfection agent.^42^ While these works all represent significant steps taken to identify NCs with conjugated molecule combinations that enhance targeting, uptake, and therapeutic outcomes, there was a lack of investigation into understanding the synergistic functionality of the conjugated components: CPPs, targeting ligands, and surface chemistry modifiers, such as PEG. Understanding which components of the designed multifunctional nanocarriers direct function, and how they direct it, will be key in guiding future NC and CPP development.

### 4.2 CPPs for enhanced transfection with NC-delivered RNA and DNA

Conjugation of specialized novel CPPs has been found to aid in RNA transfection, yielding increased expression or silencing of proteins. The PepFect/NickFect peptide family has led to some success with transfection: both PepFect6- and NickFect70-conjugated NCs loaded with siRNA successfully knocked down overexpressed genes in endometriosis.^60^ Similar results have been observed for metal–organic framework (MOF) NCs conjugated to PepFect CPPs, specifically the PepFect chimeric peptide family, PF14 and PF221.^61^ Conjugation of CPPs turned these MOFs (porous zeolitic imidazolate and graphene oxide) into viable multifunctional NCs for transfection; CPP-conjugated MOFs were taken up *via* scavenger class A internalization, and transfection of plasmids delivered in CPP-conjugated MOF NCs was increased by 2–8 fold.

Novel CPPs, which have been modified to enhance characteristics of amphiphilicity or hydrophobicity, have also found success in achieving transfection. Deshayes *et al.* investigated the use of a novel amphipathic CPP “WRAP” for NC delivery of siRNA. They found that WRAP conjugation enhanced uptake and yielded increased siRNA silencing.^62^ Sugimoto *et al.* investigated specific moieties of a PEGylated LNP-conjugated CPP that enhance cellular uptake. They found that in peptides of repeating glutamic acid (E) and lysine (K) units, one or more K or arginine groups conjugated adjacent to the spacer sequence drastically enhances cellular uptake, cytoplasmic delivery, and successful transfection of mRNA.^63^ Bazaz *et al.* developed a novel (7-octenyl)alanine and (4-pentenyl)alanine-based CPP, which had sequence-specific introductions of hydrophobicity throughout its structure, that enhanced stability and biodistribution of splice-switching oligonucleotide-based nanocomplexes, reducing systemic clearance when administered intravenously.^64^ Interestingly, the CPP modification which had the most hydrophobic modifications, hPep3, was observed to induce dose-dependent splice-switching activity after transfection at a 35-fold increase when compared to free splice-switching oligonucleotides in HeLa 705 cells.^64^ Gomes dos Reis *et al.* found that while PLGA was an excellent polymeric choice for pDNA encapsulation and NC formulation, conjugation of their novel lactoferrin-derived CPP was essential for intracellular delivery of PLGA NCs in Beas-2B and A549 cells. The CPP-PLGA-pDNA NCs utilized clathrin-mediated endocytosis, achieved endosomal escape, and successfully transfected cells, indicating that this designed carrier is a promising tool for gene delivery to the lungs.^65^

### 4.3 Outlook on arginine-rich and cationic CPPs for NA delivery

Arginine-rich and other cationic CPPs for NA NC delivery can enhance endosomal escape as well as increase cellular uptake. Additional modifications to these sequences can enhance CPPs to have added benefits for NC delivery and increase overall efficacy of treatments, such as increased hydrophobicity, to enhance transfection or novel protein conjugation for tumor-specific targeting. Arginine-rich CPPs specifically appeared to drastically enhance intracellular delivery of NA compared to unconjugated controls. However, more work is needed to understand the coaction between each component (respective CPPs, NCs, and additional surface chemistry modifications, like PEGylation). Understanding the mechanisms these novel NCs use to achieve targeted delivery and uptake will allow for future design of novel CPPs that have the specific structure and components needed to fully augment the observed transfection improvements.

## 5. CPPs for delivering protein and peptide therapeutics

Protein cargo poses a unique challenge in drug delivery in that proteins have very poor bioavailability due to their large size, hydrophilicity, and immunogenicity, particularly for oral delivery where they are degraded by digestive juices. This issue is exacerbated for protein drugs with an intracellular target, as these have poor cellular uptake. CPP conjugation has been found to enhance the cytosolic bioavailability of protein drugs, and this effect has been seen to be further enhanced through encapsulation of the therapeutic protein into a CPP–NC platform. Qian *et al.* demonstrated this in their development of a granzyme-B-loaded *p*-2-methacryloyloxy ethyl phosphorylcholine-modified hyaluronic acid (PMPC/HA) NC. When compared to CPP-granzyme-B conjugates and free granzyme-B, TAT-granzyme-B-PMPC/HA NCs had the highest tumor accumulation and cellular uptake, resulting in the most tumor reduction.^66^ NCs designed for intracellular delivery of d-amino peptides *via* a poly-arginine-derived CPP motif also enhanced cellular uptake *via* clathrin-mediated endocytosis and showed improved accumulation and uptake in non-small cell lung cancer.^67^

Similar phenomena have been observed for oral delivery systems developed to treat diabetes, with particular strides made for insulin. Conjugation of the novel cell-penetrating peptide cystine-modified protamine to insulin-loaded-polylactic acid-PEG-mesoporous silica NCs was found to reduce electrostatic interactions with mucus, decreasing mucus trapping and facilitating cellular uptake. This enhanced overall oral insulin delivery when compared to unconjugated NCs and free insulin.^68^ Using a novel alginate insulin-loaded NC, Li *et al.* found that conjugation of CPP octa-arginine enhanced the paracellular permeability of the NCs, and the developed NC as a whole exhibited properties of controlled release of the loaded insulin.^69^ While these studies represent significant strides in the development of oral NC delivery systems, more work is needed to understand why the specific CPP–NC combinations yielded enhanced NC transport through barriers, as some studies have shown that certain CPP–NC combinations can hinder nasal mucosa permeation. For example, de Souza Von Zuben *et al.* found that in TAT and penetratin insulin-loaded liposomes, electrostatic interactions between CPP and insulin decreased the percent insulin that could be encapsulated and decreased the percent nasal mucosa permeation, resulting in an overall poorer drug delivery system.^70^ This directly opposes the findings by Tan *et al.*, who found that adding the CPP to their NC was the factor that enhanced mucus permeation, not PEGylation, as might have been expected. Two different types of protein cargo, recombinant growth hormone and insulin, were found to enhance this mucus permeation.^26,68^ This highlights the need for careful consideration of the interactions between NC components when developing a CPP drug delivery system with protein cargo, particularly when the NC components themselves are also biomolecules.

Strides have also been made in CPP–NC-based delivery of proteins for treating cancer. Recent work by Liu *et al.* investigated the use of a novel multivalent CPP for enhancing delivery of ovalbumin(OVA)-loaded PLGA NCs, which they found promoted cargo lysosomal escape and cross presentation, reducing overall tumor burden and enhancing survival outcomes in E·G7-OVA lymphoma models.^71^ Additionally, Mohammadi *et al.* found that in their novel CPP–NC for protein delivery (Class I Kb-restricted peptide epitope), CPP conjugation to the nanofiber NC complex enhanced antigen presentation, inducing a significant antitumor immune response in their TC-1-grafted mouse model.^72^ Lee *et al.* found that the conjugation of novel C-terminal-acylated peptide (NH2-Ala-Arg-Arg-Cys-Val-Arg-Ala-Arg-Thr-Arg-Ac) with amphiphilic chlorin enhanced tumor penetration and successfully blocked PD-1/PD-L1 interactions and reduced PD-L1 expression.^73^ The NCs were also responsive to photodynamic therapy, and the combined effects of these treatments yielded successful immunomodulation of the tumor microenvironment of CT26 cancer, enhancing outcomes in the treatment of abscopal metastatic tumors.^73^ While each of these works represents significant strides in targeted protein therapy delivery, the versatility of outcomes and variety of targeting benefits associated with NCs conjugated with similar CPPs and cargo highlights the need for understanding the interactions between NC components, and how this affects targeting outcomes.

## 6. CPPs to improve co-delivery of multiple therapeutics

Combination therapies of a small molecule drug combined with immunomodulatory therapy, nucleic acid cargo, and/or a secondary small-molecule drug are becoming increasingly favorable as treatment regimens for highly recalcitrant diseases, such as cancer. Encapsulating combination therapies into NCs can improve treatment efficacy by increasing accumulation and uptake.^74^ One such method is the dual loading of both a chemotherapy drug and small-molecule protein inhibitors into CPP–NCs to block proteins associated with cancer cell survival and chemoresistance. Zhao *et al.* co-delivered doxorubicin with quercetin, a monocarboxylate inhibitor that limits lactate production to prevent crosstalk between tumor cells and their microenvironment. Conjugation of legumain-responsive KC26 (ke5Ne4GPTN2R9C, k: d-lysine; e: d-glutamate) peptides to the co-loaded liposome allowed selective internalization into 4T1 breast cancer cells overexpressing legumain and subsequent inhibition of lactate metabolism and angiogenesis.^75^ As lactate promotes breast cancer progression, regulation of lactate metabolism caused significant cell death compared to free drug.^75^ Liu *et al.* co-delivered doxorubicin with tariquidar, a P-glycoprotein inhibitor, using liposomes decorated with a novel peptide comprising octa-arginine incorporated into the C-terminus of an EGFR-specific targeting peptide. Their system significantly inhibited tumor growth of drug-resistant triple-negative breast cancer cells *in vitro* and *in vivo* compared to free drug, liposomes with DOX only, and liposomes without the CPP. This was a result of improved EGFR targeting and cell penetration and inhibited efflux.^76^ In both cases, incorporation of both drugs into a CPP-conjugated NC improved the response to the chemotherapeutic by increasing tumor sensitivity to doxorubicin.

Another promising approach is co-loading a chemotherapeutic and siRNA or microRNA (miRNA) to knockdown the expression of proteins critical to tumor growth and metastasis. While RNA interference (RNAi) is a potent anticancer therapy, the efficacy of free siRNA and miRNA is limited by their sensitivity to nuclease degradation in serum, poor cell uptake, and inefficient endosomal escape.^41^ Thus, encapsulation strategies that protect the nucleic acids from nucleases and improve cellular delivery—such as CPP-conjugated NCs with a cell targeting and intracellular release mechanism—are crucial. Zhao T. *et al.* synthesized a dual-pH responsive cell-penetrating peptide with R8, a polyanionic shielding domain that exerts charge shielding on the polyarginine at physiological pH, with a pH-sensitive imine linkage between them. The CPP domain also facilitates the endosomal escape of siRNA complexed with stearylated octahistidine, which dissociates in the cytosol. This multistage mechanism effectively delivers siRNA against polo-like kinase-1 (siPLK-1), a mitotic regulator overexpressed in cancer cells, and docetaxel to tumors to downregulate expression of PLK-1 and inhibit tumor growth. This multistage delivery was observed to have the greatest tumor growth reduction and lowest toxicity when compared to siPLK-1-loaded liposomes, DTX-loaded liposomes, and the two cargos delivered in separate liposomes, highlighting the significance of combined therapies enhanced with CPP–NC platforms.^77^ Huang *et al.* similarly combined small-molecule with nucleic acid cargo, developing PLGA-PEG NCs encapsulating etoposide (inactivates DNA topoisomerase II) and siRNA against PIK3CA (siPIK3CA, which inhibits PIK3CA to prevent small-cell lung carcinoma proliferation and tumor growth) and conjugated the CPP TAT externally. Co-loading in this carrier system demonstrated synergy between etoposide and siPIK3CA, as the IC_50_ (half-maximal inhibitory concentration) of carriers loaded with both components was significantly lower than that of single-loaded carriers, indicating enhanced potency. The co-loaded system had significant anti-tumor effects.^78^

In summary, multi-drug delivery has significant promise for improved outcomes of recalcitrant diseases by attacking the disease on multiple fronts. CPP–NC delivery of multi-drug therapies can enhance this efficacy further, taking therapeutics directly to their active site, thereby enhancing potency.

## 7. Conclusion and outlook

The investigation thus far of CPPs in conjugation with cargo-loaded NC systems has yielded promising results in terms of enhanced cellular uptake and therapeutic outcomes. Taking this a step further, recent works have developed novel CPPs designed for conjugation to NCs with a goal of enhanced conjugation, barrier penetration, and/or target cell population uptake. These additional functionalities have been shown to further enhance therapeutic outcomes. Despite these advances, most studies have emphasized only the impacts of CPP conjugation/novel CPP design on therapeutic outcomes. The large-scale impact is clear and an important indicator of success; however, the field would benefit from a more in-depth understanding of what is driving the success of these systems. Specifically, many of the studies cited herein failed to investigate what cellular mechanisms drove the targeting and uptake observed in CPP–NCs. An enhanced understanding of the mechanistic level of CPP–NC functionality, and how synergistic effects between system properties such as CPP structure, sequence and concentration, cargo type, and NC material properties, will allow for more informed future rational design of CPP–NCs and better understanding of why some of these formulations may fail. Further, few of the cited studies quantified or reported the peptide conjugation efficiency, degree of surface coverage, quantity of peptide present, or storage stability, all parameters required for rational design of future NC systems, as well as for optimization for clinical translation of these drug delivery vehicles. Future investigation of both of these areas, underlying mechanisms and NC characterization, will open the door for rationally designed NCs for personalized medicine, a long-standing goal of the nanotechnology field.^1^ These rationally designed CPP–NC systems could drive solutions to improve therapeutic delivery for diseases beyond cancer, including autoimmune disorders and rare and chronic diseases.

## Conflicts of interest

There are no conflicts to declare.

## Data Availability

This is a review article and no new data was generated for the work.
